# Editorial: Environmental exposures and thyroid health

**DOI:** 10.3389/fendo.2023.1154547

**Published:** 2023-02-13

**Authors:** Maaike van Gerwen, Janete Maria Cerutti, Catherine Fiona Sinclair

**Affiliations:** ^1^ Department of Otolaryngology-Head and Neck Surgery, Icahn School of Medicine at Mount Sinai, New York, NY, United States; ^2^ Institute for Translational Epidemiology, Icahn School of Medicine at Mount Sinai, New York, NY, United States; ^3^ Genetic Bases of Thyroid Tumor Laboratory, Division of Genetics, Department of Morphology and Genetics, Escola Paulista de Medicina, Universidade Federal de São Paulo, São Paulo, Brazil; ^4^ Department of Surgery, Monash University, Melbourne, VIC, Australia

**Keywords:** thyroid, environment, endocrine disruptors, thyroid cancer, thyroid function, pollutants

A variety of environmental exposures have been suggested to explain, at least in part, the increasing trends of both thyroid cancer and thyroid disease over recent decades. Thyroid cancer incidence has increased on average 3.6% per year (95% confidence interval (CI): 3.2%- 3.9%) between 1974 and 2013 in the United States (US). (1) This increase has primarily been due to an increase in papillary thyroid cancer diagnoses, which increased on average by 4.4% per year (95% CI: 4.0%- 4.7%). (1) From 2013, average subcentimeter thyroid cancer incidence rates started to decline by -3.7% per year (95% CI: -8.7%- 1.7%), while the average incidence of thyroid cancers measuring more than one centimeter increased at +2.0% per year (95% CI: 1.1%- 2.9%). (2) These increasing papillary thyroid cancer incidence rates have also been reported in other countries suggesting a worldwide phenomenon. (3)

In addition to carcinoma, studies have also shown increasing trends in autoimmune thyroid diseases. A study published in 1972 reported an increasing trend in Hashimoto’s thyroiditis, also known as chronic lymphocytic thyroiditis or autoimmune thyroiditis, from 6.5 per 100,000 persons in 1935-1944 to 69.0 per 100,000 persons in 1965-1967 in Minnesota (US). (4) Retrospective review of medical records in an Italian region showed that Hashimoto’s thyroiditis became 10 times more common between 1990 and 2005. (5) An increasing annual frequency of Hashimoto’s thyroiditis was found at a Sicilian cytological unit (Italy) between 1988 and 2007. (6) These global increasing trends in thyroid cancer and thyroid disease, which cannot be solely explained by increased access, use and quality of diagnostic tools, indicate that modifiable risk factors including exposure to environmental exposures may play a causative role (7). (8, 9) The articles published in this research topic highlight the variety of risk factors and exposures associated with thyroid health.

## Medical conditions as endogenous risk factors

Diabetes is the most commonly diagnosed endocrine disorder. (9) Although diabetes and thyroid dysfunction are closely linked conditions, diabetes has not been consistently linked to thyroid cancer. A pooled analysis of five prospective US studies (n= 674,491) showed no association between a self-reported history of diabetes and thyroid cancer risk (HR: 1.08 (95% C: 0.83- 1.40)). (10) However, the recent meta-analysis by Dong et al showed that patients with diabetes had a 1.3-fold increased risk of thyroid cancer (95% CI: 1.22- 1.44) compared to non-diabetes patients; this positive association was found for both males (RR: 1.26 (95% CI: 1.12- 1.41)) and females (RR: 1.36 (95% CI: 1.22- 1.52)). Dong et al. performed a subgroup analysis by type of diabetes and found a 1.34-fold increased risk of thyroid cancer in the population with type 2 diabetes (95% CI: 1.17- 1.53). This systematic review and meta-analysis including 20 cohort studies and more than 300,000 individuals provides evidence that the risk of thyroid cancer was increased in approximately 30% of patients with diabetes.

As previously described, the incidence of both thyroid cancer and Hashimoto’s thyroiditis has increased steadily over past decades. Although these increases may be caused by access, use and quality of diagnostic tools, chronically unresolved inflammation, as present in Hashimoto’s thyroiditis, has been associated with increased risk of malignant disease. In fact, chronic infection or chronic inflammatory states are thought to be a causal factor in approximately 20% of all human cancers. (11) Xu et al. performed a systematic literature review and meta-analysis to understand the impact of Hashimoto’s thyroiditis on the progression of papillary thyroid cancer. The meta-analysis included 39 original research articles and showed that Hashimoto’s thyroiditis is a risk factor for thyroid cancer (pooled odds ratio (OR): 1.71 (95% CI: 1.57- 1.80)). On the contrary, prevalence of extrathyroidal extension (pooled OR: 0.79 (95% CI: 0.72- 0.86)), central lymph node metastasis (pooled OR: 0.80 (95% CI: 0.74- 0.87)), distant metastasis (pooled OR: 0.52 (95%: 0.31- 0.87)), *BRAF V600E* mutations (pooled OR: 0.47 (95% CI: 0.43- 0.52)), and recurrence (pooled OR: 0.32 (95% CI: 0.18- 0.58)) were significantly lower in patients with both Hashimoto’s thyroiditis and thyroid cancer, suggesting that Hashimoto’s thyroiditis is a protective factor against PTC progression. Xu et al. thus demonstrated that Hashimoto’s thyroiditis seems to be a “double-edged sword” in thyroid cancer.

Iodine deficiency can have major thyroid-related health consequences including goiter, hypothyroidism, impaired mental function, and delayed physical development. Iodine deficiency before or just after birth has been associated with fetal and infant mortality, congenital anomalies and endemic cretinism. (12) Cretinism is caused by severe iodine deficiency *in utero* and is characterized by gross intellectual disability and varying degrees of short stature, deaf-mutism, and spasticity. (12) Li et al. performed a cross-sectional study including 31 neurological cretins and 85 controls to reassess thyroid status following iodine supplementation after birth. A significantly higher prevalence of subclinical hypothyroidism (*P*= 0.029) and thyroid nodules (*P*< 0.0001) was found in the cretin group compared to the control group, which highlights the irreversible impact of iodine deficiency *in utero* on the thyroid gland.

## Endocrine disrupting chemicals

Exposure to endocrine disrupting chemicals (EDCs) as a potential modifiable risk factor for thyroid dysfunction and thyroid cancer is of growing interest to researchers. Although exposure to endocrine disrupting chemicals (EDCs) has been associated with changes in thyroid function, the potential carcinogenic effect of EDCs on the thyroid gland remains to be evaluated, as study results are inconsistent. (13)

Iodide uptake mediated by the sodium-iodide symporter (NIS) is identified as the first limiting step involved in the production of thyroid hormones. Perchlorate, nitrate, and thiocyanate competitively inhibit the NIS-mediated iodine uptake, thus modifying iodide uptake and affecting thyroid hormone synthesis. The review by Serrano-Nascimento et al. focusses on these anions, concluding that the impact of exposure to NIS-inhibitors is still inconclusive and controversial and further evaluation is needed.

Ayhan et al. examined the association between *in utero* exposure to chlordecone, an organochlorine insecticide with endocrine disruptive properties, and thyroid function in 124 boys and 161 girls at the age of 7 years. While they found that prenatal exposure to this EDC was associated with elevated levels of thyroid stimulating hormone (TSH) in the third quartile of cord-blood chlordecone concentrations for girls compared to the lowest quartile (β_adjusted_: 0.22 (95% CI: 0.01; 0.44)), it was not associated with significant changes in free triiodothyronine (fT3) and free thyroxine (fT4) in girls and boys.

Tang et al. investigated the effect of early life exposure to triclosan, an antimicrobial chemical with potential endocrine disruptive properties, on thyroid hormone levels and histopathological changes of thyroid follicles. Zebrafish were exposed to triclosan at 0 (control), 3, 30, 100, 300, and 900 ng/mL. Increasing triclosan from 3 to 300 ng/mL reduced total triiodothyronine (TT3), fT3 and fT4 levels and induced histopathological changes within thyroid tissues.

To examine the toxic and endocrine disruptive effects of glyphosate-based herbicides in experimental models, Dal’ Bó et al. investigated the effects of Roundup^®^, a glyphosate containing herbicide, on normal and papillary thyroid carcinoma cell lines. Exposure to Roundup^®^ at acceptable occupational exposure levels (160µg/L) caused the death of 43% to 50% of the human thyroid-derived cell lines in 24 hours and 33% in 48 hours. Dal’ Bó et al. concluded that Roundup^®^ exposure presents a non-monotonic dual dose-response curve with significant cell death after low dose exposure with important proliferative effects.

Sousa-Vidal et al. assessed the effects of intrauterine exposure to di(2-ethylhexyl) phthalate (DHEP), a widely used EDC in the production of malleable plastics, on the hypothalamus-pituitary-thyroid (HPT) axis in offspring rats in adulthood. A decrease in serum TSH and T4 levels was found in female rats exposed to DHEP *in utero*, while an increase in serum TSH levels was found in exposed male rats. These results confirm the impact of intrauterine DHEP exposure on the HPT axis and the potential increased susceptibility to develop thyroid dysfunction.

In conclusion, this research topic provides a broad overview of the complex and extensive interplay of endogenous and exogenous exposures potentially involved in thyroid dysfunction and thyroid cancer. It summarizes the recent achievements in disentangling the potential roles of these exposures, thereby providing a basis for future studies to better explore and understand reasons for the increasing trend in the incidence of thyroid cancer and thyroid disease. In turn, this may allow for the development of preventative strategies, personalized management, and exposure health policies to improve thyroid health.

## Author contributions

MG, JC and CS contributed to conception and design of the study. MG wrote the first draft of the manuscript. All authors contributed to manuscript revision, read, and approved the submitted version.
